# A yeast-based assay identifies drugs that interfere with immune evasion of the Epstein-Barr virus

**DOI:** 10.1242/dmm.014308

**Published:** 2014-02-20

**Authors:** Cécile Voisset, Chrysoula Daskalogianni, Marie-Astrid Contesse, Anne Mazars, Hratch Arbach, Marie Le Cann, Flavie Soubigou, Sébastien Apcher, Robin Fåhraeus, Marc Blondel

**Affiliations:** 1 Institut National de la Santé et de la Recherche Médicale UMR 1078; Université de Bretagne Occidentale, Faculté de Médecine et des Sciences de la Santé; Etablissement Français du Sang (EFS) Bretagne; CHRU Brest, Hôpital Morvan, Laboratoire de Génétique Moléculaire, Brest F-29200, France.; 2 Cibles Thérapeutiques, Institut National de la Santé et de la Recherche Médicale UMRS940, Institut de Génétique Moléculaire, Université Paris 7, Hôpital St Louis, F-75010 Paris, France.

**Keywords:** EBV-associated cancers, Cell-based drug screening, EBNA1 GAr domain, Yeast-based models, Immune evasion, Doxorubicin, Daunorubicin, 5-fluorouracil

## Abstract

Epstein-Barr virus (EBV) is tightly associated with certain human cancers, but there is as yet no specific treatment against EBV-related diseases. The EBV-encoded EBNA1 protein is essential to maintain viral episomes and for viral persistence. As such, EBNA1 is expressed in all EBV-infected cells, and is highly antigenic. All infected individuals, including individuals with cancer, have CD8^+^ T cells directed towards EBNA1 epitopes, yet the immune system fails to detect and destroy cells harboring the virus. EBV immune evasion depends on the capacity of the Gly-Ala repeat (GAr) domain of EBNA1 to inhibit the translation of its own mRNA in *cis*, thereby limiting the production of EBNA1-derived antigenic peptides presented by the major histocompatibility complex (MHC) class I pathway. Here we establish a yeast-based assay for monitoring GAr-dependent inhibition of translation. Using this assay we identify doxorubicin (DXR) as a compound that specifically interferes with the GAr effect on translation in yeast. DXR targets the topoisomerase-II–DNA complexes and thereby causes genomic damage. We show, however, that the genotoxic effect of DXR and various analogs thereof is uncoupled from the effect on GAr-mediated translation control. This is further supported by the observation that etoposide and teniposide, representing another class of topoisomerase-II–DNA targeting drugs, have no effect on GAr-mediated translation control. DXR and active analogs stimulate, in a GAr-dependent manner, EBNA1 expression in mammalian cells and overcome GAr-dependent restriction of MHC class I antigen presentation. These results validate our approach as an effective high-throughput screening assay to identify drugs that interfere with EBV immune evasion and, thus, constitute candidates for treating EBV-related diseases, in particular EBV-associated cancers.

## INTRODUCTION

The Epstein-Barr gammaherpesvirus (EBV) was the first oncogenic virus described in humans (Epstein et al., 1964; Thorley-Lawson and Allday, 2008; Young and Rickinson, 2004). EBV is a ubiquitous virus that infects over 90% of the human population. Primary infection with EBV is normally asymptomatic, but can cause in teenagers and adults a self-limiting lymphoproliferative disease termed infectious mononucleosis. In most individuals it persists as a lifelong latent asymptomatic infection but, in individuals suffering from some forms of immune suppression (e.g. grafted patients or HIV-infected people), EBV can cause severe lymphoproliferative disorders. EBV is also linked to certain types of cancer, the best known of which is the endemic form of Burkitt’s lymphoma for which malaria infection has been proposed as a cofactor (Thorley-Lawson and Allday, 2008). Another example is nasopharyngeal carcinoma, which has a high prevalence among men in South-East China and carries EBV in virtually all cases. Other cancers linked to EBV include Hodgkin’s lymphoma and some gastric cancers (Hsu and Glaser, 2000; Young and Rickinson, 2004). EBV and its link to human diseases have been known for nearly 50 years but, as of today, there is no specific treatment against EBV-associated diseases, in particular cancers.

The presentation of endogenous peptides on major histocompatibility complex (MHC) class I molecules allows the immune system to distinguish between self and non-self. The presence of viral epitopes on MHC class I molecules serves as a signal for CD8^+^ T cells to detect and destroy infected cells. Hence, several viruses, in particular those with a latent phase, have evolved various strategies to prevent peptide display by MHC class I molecules. The EBV-encoded EBNA1 protein is expressed in all types of EBV-infected cells and is the only viral protein detected in Burkitt’s lymphoma cells (Rowe et al., 1987). EBNA1 is a genome-maintenance protein (GMP) (Blake, 2010) that is essential to maintain viral episomes and for viral persistence, but, despite its strong antigenic potential, the immune system does not detect and destroy EBNA1-expressing cells (Blake et al., 1997; Rickinson and Moss, 1997). This stealthiness of EBNA1 is due to the inhibition of *EBNA1* mRNA translation via a *cis*-acting mechanism mediated by a glycine-alanine repeat sequence (GAr domain) that consists of a stretch of single alanines separated by one, two or three glycines located in the N-terminal part of EBNA1 (Levitskaya et al., 1995). Hence, the GAr domain controls the translation of its own mRNA (Starck et al., 2008; Yin et al., 2003), thereby minimizing the production of peptide substrates, the so-called defective ribosomal products (DRiPs) or pioneer translation products (PTPs), for the MHC class I pathway (Apcher et al., 2011; Apcher et al., 2012; Fåhraeus, 2005; Yewdell, 2011). This capacity prevents CD8^+^ T cells from detecting EBNA1-expressing cells. In conjunction with a long EBNA1 half-life, the function of GAr on antigenic peptide production constitutes an efficient means by which the virus can evade the immune system whilst expressing EBNA1 at a functional level. The effect of the GAr domain is ‘length-dependent’, meaning that its length determines its efficacy: a longer domain displays a stronger inhibitory effect on both mRNA translation and antigen presentation (Apcher et al., 2009).

**TRANSLATIONAL IMPACT**

**Clinical issue**

Epstein-Barr virus (EBV), a ubiquitous virus that infects more than 90% of the human population, is intimately linked to several different human cancers, including Burkitt’s lymphoma, Hodgkin’s lymphoma and nasopharyngeal carcinoma, which is one of the most frequent cancers in South-East China. Like several other viruses with a latent phase, EBV has evolved a strategy to evade the immune system by interfering with peptide display on major histocompatibility complex (MHC) class I molecules. In the case of EBV, this immune-evasion strategy is based on inhibition of translation of the mRNA that encodes EBV nuclear antigen-1 (EBNA1) by the EBNA1 Gly-Ala repeat (GAr) domain. Thus, EBNA1 itself suppresses the production of EBNA1-derived antigenic peptide substrates. Currently, there is no specific treatment against EBV-related diseases, but interference with EBV stealthiness represents an interesting opportunity for the development of novel immune-based therapeutics that selectively target EBV-carrying cells, including EBV-carrying cancer cells.

**Results**

In this study, the authors establish an original yeast-based model that recapitulates all the features of GAr-mediated suppression of *EBNA1* mRNA translation. They use their model to identify doxorubicin as a compound that specifically interferes with GAr-mediated suppression of translation. They show that the effect of doxorubicin on GAr-mediated translation control is independent of its genotoxic effect and that doxorubicin and its active analogs stimulate EBNA1 expression in mammalian cells in a GAr-dependent manner. Finally, the authors demonstrate that doxorubicin and its active analogs overcome the GAr-dependent restriction of MHC class I antigen presentation in mammalian cells.

**Implications and future directions**

These results validate the yeast-based assay developed by the authors as an effective high-throughput cell-based screening approach to identify compounds that specifically interfere with EBV immune evasion, thereby rendering EBV-carrying cells targets for the immune system. Notably, these findings identify a class of compounds already in clinical use for other applications as potent suppressors of EBV immune evasion. These drugs could constitute candidates for the treatment of EBV-related diseases, in particular EBV-associated cancers. More generally, these findings suggest that it might be possible to target the translation of at least some specific viral mRNAs for therapeutic intervention.

It is still not clear how the GAr domain mediates inhibition of its own mRNA translation in *cis*. Two hypotheses are currently being explored. In the first, the nascent GAr peptide would inhibit initiation of translation of its own mRNA by a specific but as-yet-unknown mechanism (Apcher et al., 2010; Apcher et al., 2009; Yin et al., 2003). According to the second hypothesis, the GAr-encoding mRNA sequence could be responsible for inhibiting translation. Indeed, it has been observed that the protein-coding sequences of many herpesviruses, including EBV, are enriched in purines (Cristillo et al., 2001), suggesting that the purine bias might be related to the immune evasion by causing the ribosome to stall and pre-terminate the elongation process (Tellam et al., 2008; Tellam et al., 2012). This model is unlikely, however, because modifying the requirement of translation initiation factors by introducing the *c-myc* internal ribosome entry site (IRES) in the non-coding 5′ untranslated region (UTR) of *EBNA1*, while keeping the coding sequence unchanged, is sufficient to overcome the GAr-mediated inhibition of both mRNA translation and antigen presentation (Apcher et al., 2010; Apcher et al., 2009). In addition, pre-terminated EBNA1 translation products are not detected in EBV-infected cells, further supporting the notion that the ribosome does not stall while reading through the GAr-encoding sequence. Furthermore, the Papio-virus-derived EBNA1 homolog is equally rich in purines but carries a serine in every eighth residue and this sequence has no effect on mRNA translation (Apcher et al., 2010).

Importantly, whatever the exact mechanism of GAr-mediated translation inhibition, the efficient T-cell response against EBV-infected cells in which the GAr domain of EBNA1 has been deleted demonstrates the importance of this domain in the viral strategy to defer antigen presentation (Apcher et al., 2010; Levitskaya et al., 1995; Yin et al., 2003). This offers a window of opportunity for therapeutic intervention aimed at disrupting the mechanisms of action of the GAr domain and thereby increasing the presentation of EBNA1-derived antigenic peptides through the MHC class I pathway. This forms the basis for an original immune-based therapeutic strategy to selectively target EBV-infected cells, including EBV-carrying tumor cells.

The versatile genetic flexibility of the budding yeast *Saccharomyces cerevisiae* and the high degree of conservation between yeast and mammalian cellular processes have made *S. cerevisiae* an invaluable tool for modeling human diseases (Bach et al., 2003; Bach et al., 2006; Bilsland et al., 2013; Blondel, 2012; Couplan et al., 2011; Mager and Winderickx, 2005; Perocchi et al., 2008), as well as for identifying and characterizing cellular pathways involved in these disorders and thereby new therapeutic targets (Giorgini et al., 2005; Louie et al., 2012).

In this study, we established a yeast-based model that recapitulates all the features of the GAr-mediated *cis*-inhibition of mRNA translation. Using this model, we isolated drugs that interfere with both *EBNA1* mRNA translation control and immune evasion. Hence, our results show that the cellular pathways involved in the GAr-mediated inhibition of translation are conserved from yeast to human and validate our yeast-based approach as a method for identifying compounds interfering with the ability of EBV to evade the immune system. Such compounds could constitute new therapeutic avenues to specifically treat EBV-related diseases, in particular EBV-associated cancers.

## RESULTS

### Development of a yeast-based model for the GAr-mediated inhibition of translation

**Fig. 1.**
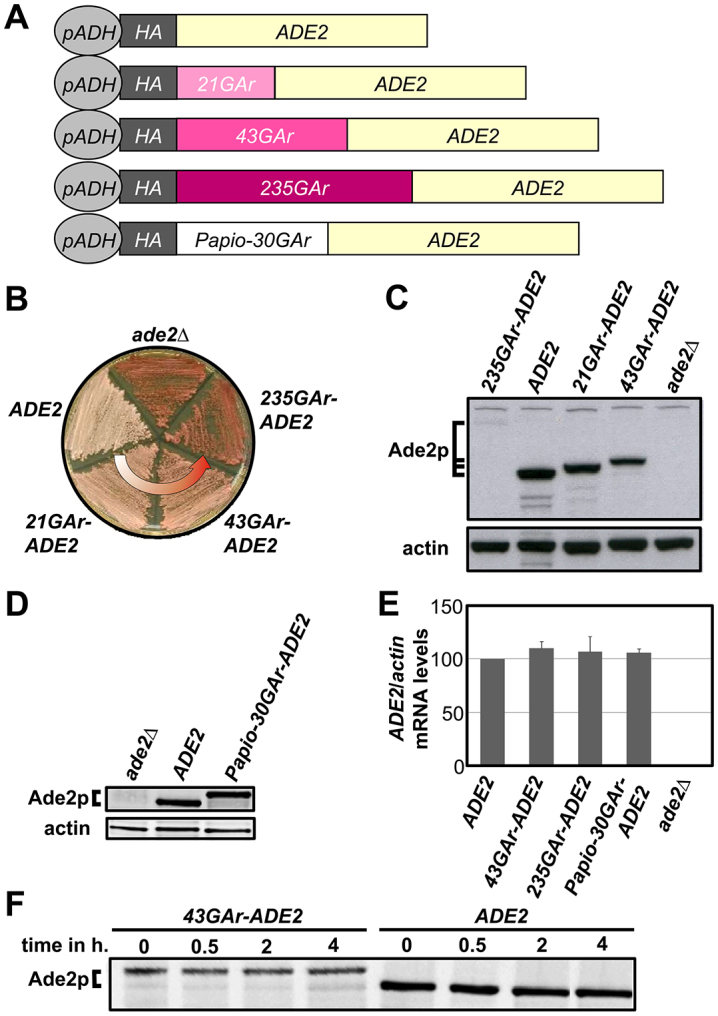
**Development of a yeast-based model for the GAr-mediated inhibition of translation.** (A) Fusions between sequences encoding GAr domains of different lengths and the *ADE2* gene are depicted. The constructs are N-terminally HA-tagged and expressed from the constitutive *ADH* promoter. (B) Clones of the *ade2Δ* strain expressing, or not, the various constructs depicted in A were streaked on glucose-rich medium and incubated for 5 days at 29°C. (C,D) SDS-PAGE and western blot analysis of protein extracts from the cells used in B. (E) qRT-PCR was used to determine the relative levels of mRNA expressed from the indicated constructs, which are shown as compared to the relative level of *actin* mRNA from the same samples. Mean data from three independent experiments are shown with s.d. (F) Half-life of the HA-Ade2p and HA-43GAr-Ade2p fusion proteins was determined by cycloheximide chase. Representative data from three independent experiments are shown.

EBNA1 has been expressed in yeast, in particular to study the crucial role of EBNA1 in EBV episome maintenance (Heessen et al., 2003; Kapoor and Frappier, 2003; Kapoor et al., 2001). These studies show that a functional EBNA1 protein can be expressed in yeast and indirectly suggest that the GAr domain might affect mRNA translation in this unicellular organism. Therefore, we hypothesized that a broad approach using yeast genetics would be suitable to identify modulators (either drugs or genes) that could interfere with the GAr-mediated inhibition of translation. To this aim, we set up a yeast-based model for the GAr-dependent inhibition of translation. Our model is based on the use of the yeast *ADE2* reporter gene, which encodes phosphoribosylaminoimidazole carboxylase (AIR carboxylase), an enzyme involved in the adenine biosynthesis pathway. Yeast cells in which the *ADE2* gene has been deleted (*ade2Δ* strain) form red colonies on rich medium, owing to the accumulation of phosphoribosylaminoimidazole (AIR), the Ade2p enzyme substrate, which becomes red when oxidized by active respiration. In contrast, cells expressing a sufficient amount of the Ade2p enzyme form white colonies. Any intermediate amount of Ade2p leads to pink colonies, whose color intensity is proportional to the Ade2p level. Starting from an *ade2Δ* strain, we first determined that the *ADH* promoter allowed for minimal expression of the *ADE2* gene that leads to white colonies, thereby ensuring that any inhibitory effect on translation of *ADE2* mRNA, even subtle, could be detected by changes in colony color. Using the *ADH* promoter, we then tested the effect of N-terminal fusion of GAr domains of various lengths to the Ade2p protein. The various constructs were N-terminally HA-tagged to allow their detection (Fig. 1A). We observed a clear GAr-length-dependent decrease in Ade2p levels, as evidenced by the white-to-red color gradient (Fig. 1B), which was confirmed by western blot analysis (Fig. 1C). This effect is promoter-independent because full-length GAr domain (235GAr) also led to the formation of dark pink colonies and to a clear decrease in the steady-state level of Ade2p when expressed from the strongest *TEF* promoter (supplementary material Fig. S1A–C). Taken together, these results suggest that, as in mammalian cells, EBNA1’s GAr domain also affects mRNA translation in a length-dependent manner in yeast. To further compare the GAr-mediated effect on protein expression in yeast and in mammalian cells, we used the GAr domain of a simian EBV-related virus that was shown to have no effect on translation in mammalian cells (Apcher et al., 2010; Apcher et al., 2009). The lymphocrypto-Papio virus infects Old World primates and expresses an EBNA1 homolog that carries short GAr-like sequences that have previously been shown to be unable to prevent antigen presentation (Blake et al., 1997). EBV’s GAr domain consists of single alanine residues separated by one, two or three glycines, whereas the GAr domain of the Papio virus EBNA1 protein contains four single serine residues inserted every seven residues of the repeat. Contrary to all EBV GAr domains tested, including the shorter 21GAr, a 30-amino-acid Papio GAr sequence was shown to have no effect on mRNA translation and antigen presentation (Apcher et al., 2010). We fused the same 30-amino-acid Papio GAr sequence to Ade2p and found that it had no effect on Ade2p levels, as compared with control cells expressing the *ADE2* gene from the same *ADH* promoter (Fig. 1D), which is in good agreement with the fact that yeast cells expressing the Papio 30GAr-Ade2p fusion protein form white colonies similar to cells expressing Ade2p (supplementary material Fig. S1D). Taken together, these results underscore that the GAr-dependent effect on protein expression operates in yeast and that the cellular mechanism(s) involved is conserved from yeast to human.

The protein level can be disturbed by affecting: (i) mRNA levels; (ii) mRNA translation; or (iii) protein stability. To determine whether the GAr domain affects its own mRNA level, we measured the expression of several constructs in the *ade2Δ* strain by quantitative real-time PCR (qRT-PCR). As shown in Fig. 1E, the *ADE2* mRNA levels obtained from the *ADH* promoter were similar in the absence or in the presence of a 43- or a 235-amino-acid GAr domain or with the Papio GAr domain, indicating that the GAr domain has no effect on the level of the *ADE2* transcript. We next determined the effect of the GAr domain on Ade2p protein stability. Using cycloheximide chase, we found that Ade2p is a very stable protein and that the GAr domain does not modify its stability (Fig. 1F). Taken together with metabolic labeling of newly synthesized proteins and pulse-chase experiments (supplementary material Fig. S2), these results demonstrate that the GAr domain inhibits translation of its own mRNA in a length-dependent manner in yeast, as it does in human cells.

### Flowchart of the drug screening assay based on the combination of the yeast model and a T-cell assay to monitor antigen presentation

**Fig. 2.**
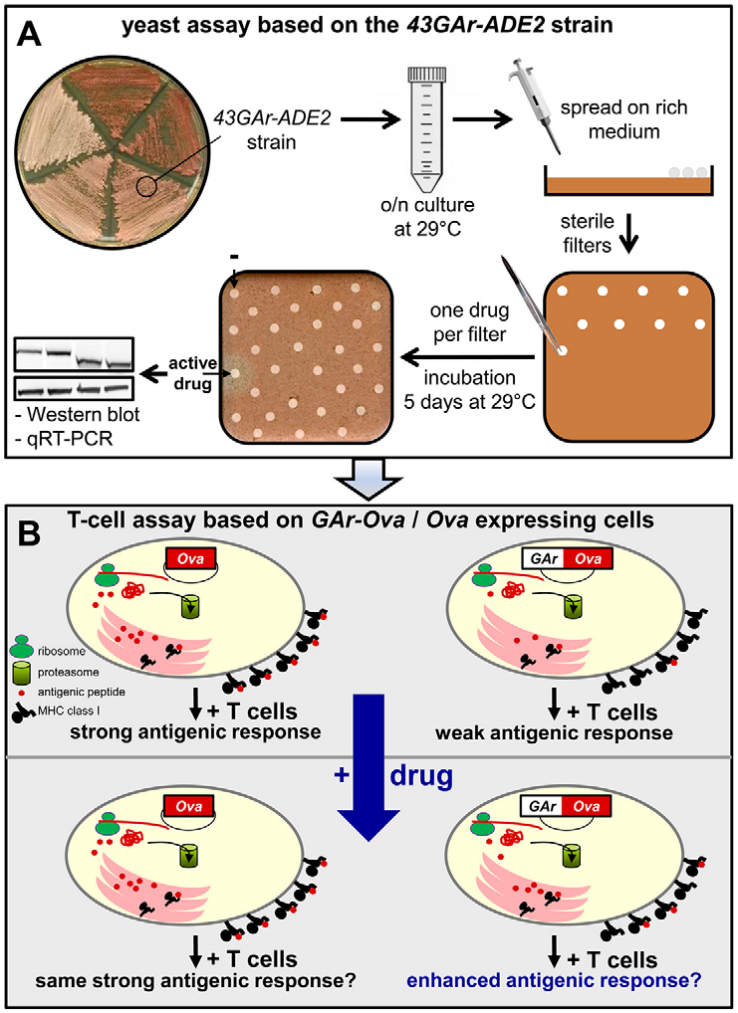
**Flowchart of the screening procedure.** (A) The yeast-based assay. The *ade2Δ* strain expressing the *HA-43GAr-ADE2* construct was cultured overnight in a liquid rich medium and spread onto a solid rich medium. Small sterile filters were then placed on the agar surface and compounds from the chemical libraries were added to the filters. Plates were incubated for 5 days at 29°C. A filter surrounded by a whiter halo indicates a compound leading to an increase in the steady-state level of HA-43GAr-Ade2p protein. The effect of this compound was then analyzed by SDS-PAGE, western blot and qRT-PCR. Of note, standard false positives obtained when using the white/red *ADE2*-based assay are compounds that interfere with oxidative phosphorylation, because active respiration is required for oxidation of AIR into a red pigment. These compounds are easily detected because they prevent yeast cell growth on non-fermentable substrate such as glycerol or ethanol. (B) The T-cell assay. The effect of a compound was analyzed using a T-cell assay. Briefly, HEK 293T Kb cells stably expressing the murine Kb MHC class I molecule were transfected with plasmids encoding Ova alone (left) or the full-length 235GAr domain fused to the N-terminus of Ova (right). Cells were then treated, or not, for 24 hours by compounds. The compounds were removed and antigen presentation was estimated using the B3Z CD8^+^ T-cell hybridoma specific for the SL8 epitope of Ova. This approach allows the identification of compounds specifically interfering with GAr-dependent control of antigen presentation.

We next took advantage of our yeast model and of the convenient white/red colony color reporter assay to screen chemical libraries for compounds that could interfere with the GAr effect on translation. The rationale is that compounds that can prevent the GAr-mediated inhibition of translation in *cis* might prevent EBV-infected cells from evading the immune system and might therefore constitute leads with therapeutic potential. On the other hand, compounds that exacerbate the inhibitory effect of the GAr domain are also interesting because EBNA1 is required for viral replication and also presents oncogenic and anti-apoptotic activities (Gruhne et al., 2009; Kennedy et al., 2003; Wilson et al., 1996). We chose the 43GAr fusion because it has an intermediate effect, yielding pink colonies on rich medium. Yeast cells were spread on a solid rich medium and exposed to filters spotted with the compounds (Fig. 2A). Hence, compounds could be isolated that either prevent the GAr effect on translation (yielding halos of whiter colonies around the filters) or exacerbate it (inducing halos of red or dark pink colonies around the filters). The advantage of this method is that, in one simple experiment, it allows numerous compounds to be tested across a large range of concentrations owing to the diffusion of the drugs into the surrounding solid medium. In addition, this design also improves the sensitivity of the screen because many compounds are toxic at high concentrations. Standard false positives obtained when using the white/red *ADE2*-based assay are compounds that interfere with oxidative phosphorylation, because active respiration is required for oxidation of AIR into a red pigment. These compounds are easily detected because they prevent yeast cell growth on non-fermentable substrate such as glycerol or ethanol. The effect of the selected compounds on *ADE2* expression was then determined by qRT-PCR and western blot analysis. The compounds that specifically affected the expression of *43GAr-ADE2* were next tested for their effect on GAr-Ova (ovalbulin) and EBNA1 expression in mammalian cells by western blotting and on MHC-class-I-restricted antigen presentation using a T-cell reporter assay (Fig. 2B, and see further below).

### Identification of drugs that interfere with the GAr-dependent inhibition of translation

**Fig. 3.**
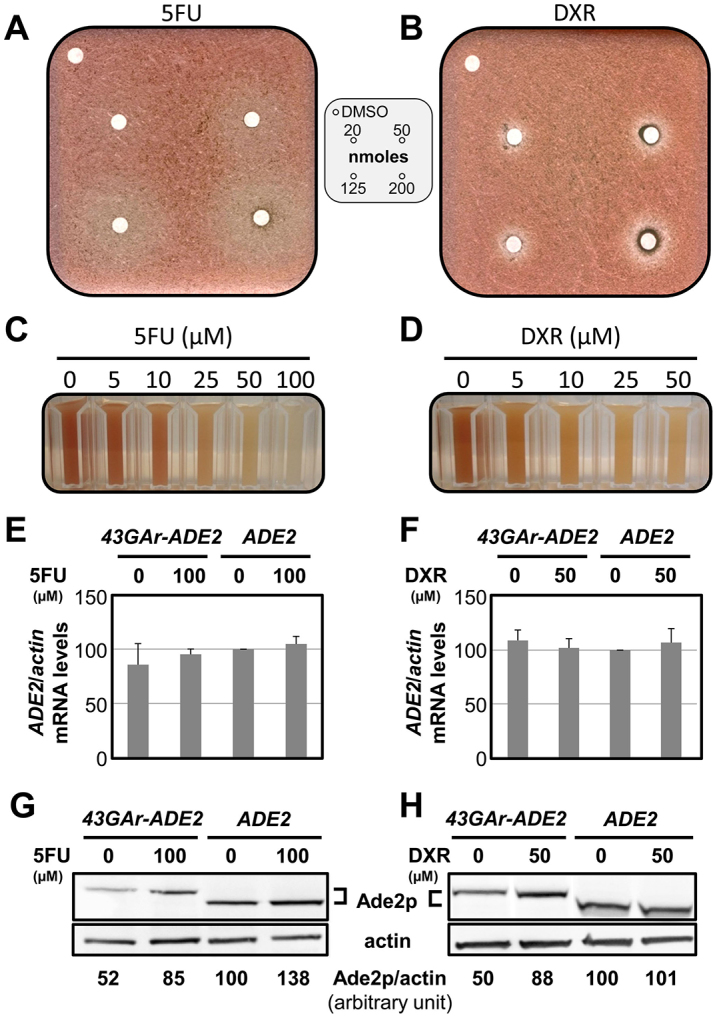
**DXR specifically interferes with the GAr-domain effect on translation in yeast.** (A,B) The *ade2Δ* strain expressing the *HA-43GAr-ADE2* construct was spread onto a rich medium and increasing concentrations of 5-fluorouracil (5FU) (A) or doxorubicin (DXR) (B) were loaded onto the filters. (C,D) Liquid cultures of exponentially growing cells of the same strain treated with the indicated concentrations of 5FU or DXR are shown. (E,F) The relative levels of *HA-ADE2* or *HA-43GAr-ADE2* mRNA in the *ade2Δ* strain treated, or not, by the indicated concentrations of 5FU or DXR were determined using qRT-PCR. Mean data from three independent experiments are shown with s.d. (G,H) The same extracts were analyzed by SDS-PAGE and western blot to determine the levels of 43GAr-Ade2p or Ade2p relative to actin, which was used as a loading control. The effect of the indicated concentrations of 5FU or DXR on HA-43GAr-Ade2p level compared with HA-Ade2p was shown. The figure shows representative data from three independent experiments.

We screened the Prestwick® and the BIOLMOL® chemical libraries, two collections of drugs for which bioavailability and toxicity studies have already been carried out in humans; therefore, isolated active compounds could directly enter drug optimization programs. Of the ~2000 drugs tested, only two were identified (corresponding to ~0.1%) that yielded a whiter halo corresponding to a clear increase in Ade2p levels, indicating that the screening assay was specific and stringent. The compounds identified were 5-fluorouracil (5FU, Fig. 3A,C) and doxorubicin (DXR, Fig. 3B,D). Moreover, both the intensity of the color and the diameter of the halo were proportional to the quantity of drug loaded (Fig. 3A–D). We further checked that 5FU and DXR did not significantly affect the mRNA levels of the various constructs by qRT-PCR (Fig. 3E,F). In order to determine whether 5FU and DXR interfere with the ability of GAr to inhibit translation, we determined the effect of these compounds on the steady-state level of 43GAr-Ade2p and Ade2p proteins (Fig. 3G,H). Both drugs led to a small but clear increase in 43GAr-Ade2p protein levels, corroborating their ability to induce a halo of whiter colonies in the yeast assay. However, only DXR exhibited a GAr-dependent effect because it had no effect on Ade2p protein level, contrary to 5FU, which also led to an increase in Ade2p protein level. These results suggest that DXR specifically affects the GAr-domain-dependent translation inhibition, whereas 5FU might have a general effect on translation.

### DXR interferes with GAr-mediated suppression of antigen presentation and translation inhibition

**Fig. 4.**
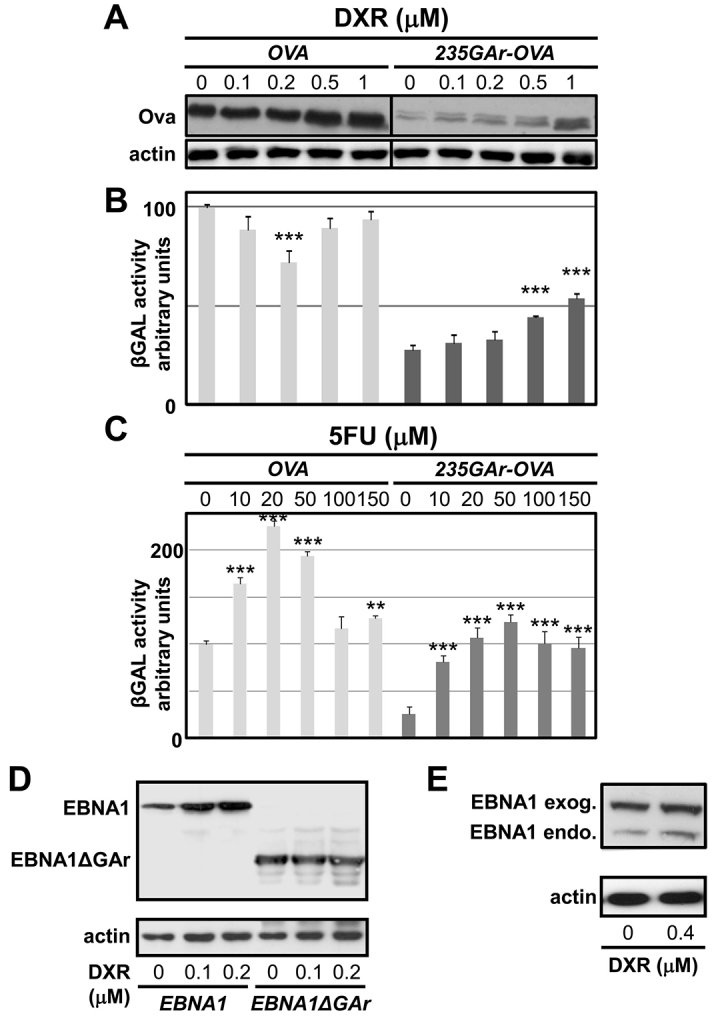
**DXR specifically stimulates antigen presentation from the 235GAr-Ova fusion construct in a T-cell assay and increases EBNA1 protein levels.** (A–C) HEK 293T Kb cells stably expressing the murine MHC class I Kb molecule were transfected with plasmids encoding chicken ovalbumin (*OVA*) alone (left) or fused to the full-length GAr (*235GAr-OVA*) (right). (B) Ova-derived MHC-class-I-restricted antigen presentation was estimated after treating cells with increasing amounts of DXR for 24 hours and measuring β-Gal activity in reporter B3Z CD8^+^ T hybridoma cells. (C) The effect of 5FU on antigen presentation was estimated as in B. Data were normalized as relative to presentation of the exogenous SL8 peptide. (A) The expression of Ova, 235GAr-Ova and actin protein levels were determined by SDS-PAGE and western blot under the same conditions. (D) Similarly, EBNA1 or EBNA1ΔGAr protein levels in HEK 293T Kb cells were determined after 24 hours of treatment with the indicated concentrations of DXR. (E) Raji Burkitt’s lymphoma cells expressing endogenous EBNA1 with a shorter GAr sequence were transfected with a cDNA encoding EBNA1, allowing the detection of endogenous and exogenous EBNA1 in the same cells after treatment with DXR for 8 hours. Actin was used as a loading control. Results were compared with untreated cells by one-way analysis of variance with Dunnett’s multiple comparison test (***P<0.001; **P<0.01).

**Fig. 5.**
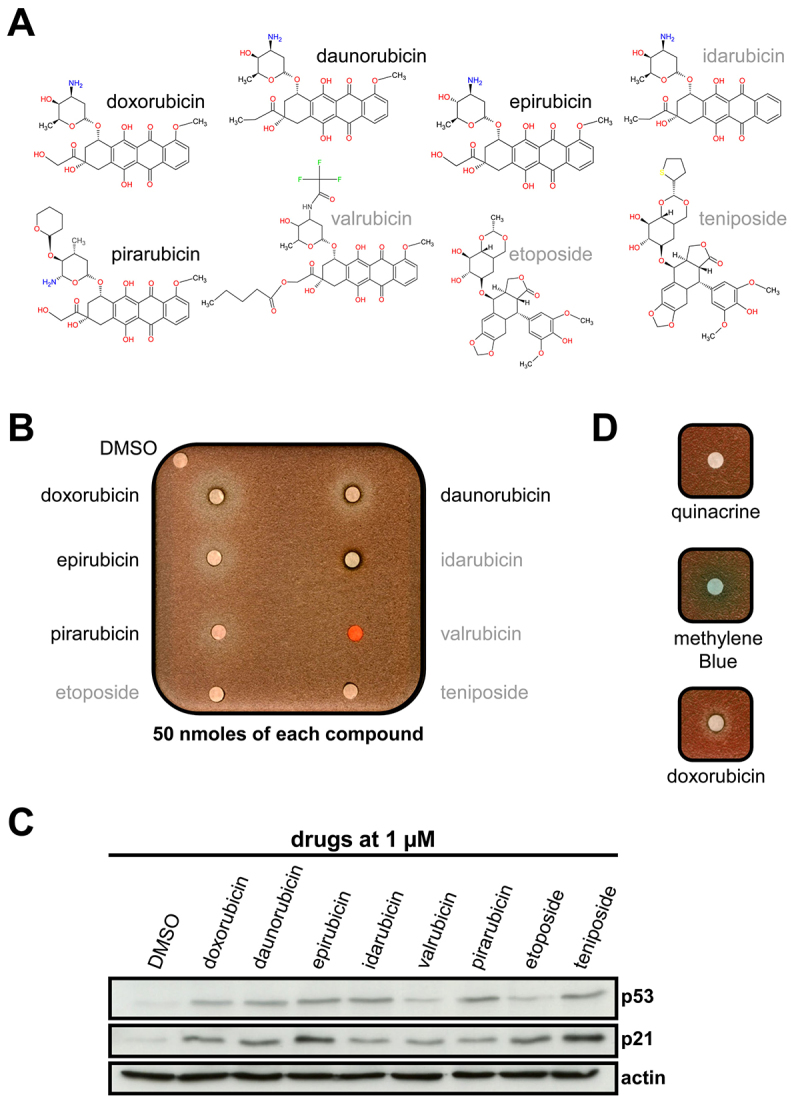
**Effect of DXR and its chemical analogs on GAr-mediated suppression of translation in yeast, and on *p53* and *p21* induction in mammalian cells.** (A) Chemical structures of doxorubicin and its analogs. (B) The effect of DXR and various chemical analogs on GAr-mediated translation was determined in the yeast assay as described in Fig. 2A and Fig. 3. (C) The genotoxic effect of DXR and its indicated analogs was estimated by western blot using as readouts the activation of the *p53* tumor suppressor gene and its downstream gene target *p21* in A549 cells. (D) The effect of two other DNA or RNA intercalating agents was determined in the yeast assay as described in Fig. 2A and Fig. 3. 20 nmoles of each compound were loaded on filters.

Having identified and validated DXR and 5FU in yeast, we next assessed the ability of these drugs to interfere with the GAr-mediated suppression of antigen presentation using a T-cell assay (Fig. 2B) (Apcher et al., 2010; Karttunen et al., 1992). For this purpose, we used a 235-amino-acid GAr domain (235GAr) fused to the N-terminus of chicken ovalbumin (Ova). Ova contains the SIINFEKL antigenic peptide sequence (SL8) that is detected by specific CD8^+^ reporter T cells (B3Z cells) when presented on murine MHC class I Kb molecules (Karttunen et al., 1992). As a control, we used Ova alone. The cDNAs encoding these polypeptides were transfected in HEK 293T cells that stably express the murine MHC class I Kb molecule (HEK 293T Kb). Transfected HEK 293T Kb cells were then treated, or not, with various concentrations of DXR or 5FU for 24 hours. Next, the drugs were removed and HEK 293T Kb cells were mixed with an equal number of B3Z cells and incubated overnight. The amount of presented SL8 peptides was then indirectly determined by measuring the β-Gal activity in B3Z cells, which is proportional to the activation of T-cell receptors specific for the SL8 peptide. This allowed us to monitor the effect of drugs on antigen presentation from the Ova and the 235GAr-Ova constructs. Both DXR (Fig. 4B) and 5FU (Fig. 4C) significantly increased antigen presentation from the 235GAr-Ova construct. However, and as observed in the yeast-based assay, this effect was GAr-specific only for DXR, because this drug did not increase antigen presentation of Ova alone. In contrast, 5FU increased antigen presentation of Ova to a similar extent as for 235GAr-Ova. We also determined by western blot the protein levels of Ova and 235GAr-Ova in HEK 393T Kb cells, and found that DXR led to a significant and dose-dependent increase in the steady-state level of GAr-Ova, whereas it had no significant effect on Ova alone (Fig. 4A), similar to what was observed in yeast. Taken together, these results show that DXR affects, in a GAr-dependent manner, both GAr-Ova expression and antigen presentation. They also validate our yeast-based drug-screening assay.

### DXR leads to a GAr-dependent increase of EBNA1 levels

Next, we determined the effect of DXR on EBNA1 protein levels. HEK 293T Kb cells expressing either EBNA1 or EBNA1ΔGAr were treated, or not, with increasing concentrations of DXR for 24 hours. As shown in Fig. 4D, DXR led to a dose-dependent increase in the steady-state level of EBNA1, whereas it had no effect on the level of EBNA1ΔGAr. The effect of DXR on the expression of endogenous EBNA1 was determined using Raji cells, which are EBV-carrying Burkitt’s lymphoma cells. Endogenous EBNA1 in Raji cells migrates faster than exogenous EBNA1, owing to its shorter GAr sequence. The levels of endogenous and exogenous EBNA1 proteins expressed in the same cells were both shown to increase following an 8-hour treatment with 400 nM DXR (Fig. 4E). Taken together, these results show that, in addition to its GAr-dependent effect on GAr-Ova expression and antigen presentation, DXR also leads to a GAr-dependent increase in the steady-state level of full-length EBNA1, either overexpressed or endogenously expressed in EBV-infected cells.

### DXR effect on GAr-mediated translation control is DNA-damage-independent

Based on the observation that DXR, but not 5FU, specifically overcomes the effect of GAr on translation, we tested commercially available chemical analogs of DXR (chemical structures depicted in Fig. 5A) in the yeast-based assay in order to further validate this compound and to determine whether a link exists, or not, between the known genotoxic activity of DXR and its effect on translation inhibition by the GAr domain. As shown in Fig. 5B, daunorubicin, epirubicin and pirarubicin were also active in the yeast assay, although to a lower extent than DXR. However, idarubicin and valrubicin, which are also close to DXR, were inactive, indicating a yet-unknown specific effect of DXR and its active analogs on the GAr-dependent inhibition of translation. Of note, budding yeast is known to be poorly permeable to a number of drugs, probably owing to the fact that, in addition to a plasma membrane, yeast cells also have a cell wall. However, it is unlikely that this could account for the lack of effect using idarubicin and valrubicin because all the tested DXR derivatives are very similar and because idarubicin resulted in a halo of toxicity around the filter where it was loaded that was bigger than the one obtained for both DXR and daunorubicin. DXR and its five analogs are all reported to cause DNA damage owing to their ability to target topoisomerase-II–DNA complexes (Nitiss, 2009). In line with this genotoxic effect, we found that they activate the p53 tumor suppressor pathway, as evidenced by their ability to induce expression of both the *p53* and *p21* genes (Fig. 5C). Of note, both etoposide and teniposide, which represent a different class of topoisomerase-II-targeting compounds, have no effect on GAr-dependent translation control in the yeast assay, whereas they activate the p53 pathway equally well as DXR and its analogs (Fig. 5B,C). These results indicate that the effect of DXR on GAr-mediated inhibition of translation is uncoupled from its already known genotoxic effect. DXR and its chemical derivatives were also described as DNA intercalating agents. In order to test whether this property could explain their effect on GAr, we tested methylene blue and quinacrine, two other DNA or RNA intercalating agents, and found them inactive (Fig. 5D). We also observed that treatment with DXR for 16 hours alters the ribosomal profile, with similar reductions in Ova and Ova-GAr mRNAs associated with ribosome, presumably owing to the inhibitory effect of DXR on DNA polymerase I (supplementary material Figs S3, S4).

**Fig. 6.**
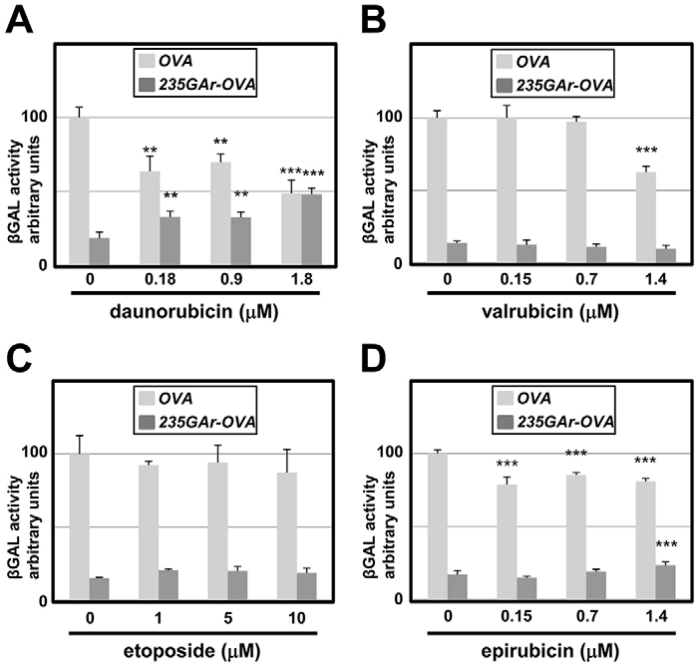
**Effect of DXR and its chemical analogs on antigen presentation in T-cell assays.** (A-D) The effect of daunorubicin, valrubicin, etoposide and epirubicin on antigen presentation was evaluated in the T-cell assay as described in Fig. 4. Results were compared with untreated cells by one-way analysis of variance with Dunnett’s multiple comparison test (***P<0.001; **P<0.01).

To further validate this structure-activity relationship (SAR), we tested the effect of some of the chemical analogs of DXR in the mammalian T-cell assay. In agreement with its effect in yeast, daunorubicin treatment led to a significant increase in antigen presentation from the 235GAr-Ova construct (Fig. 6A). By contrast, and as in the yeast assay, both valrubicin (Fig. 6B) and etoposide (Fig. 6C) had no effect. Epirubicin, which is moderately active in the yeast assay, had a modest effect in the T-cell assay (Fig. 6D). Taken together, these observations suggest that our yeast-based assay can be used to perform SAR studies for compounds that interfere with the GAr-domain effect on translation. In addition, based on the comparison between the effects on DNA damage and on overriding the GAr-dependent translation suppression, these results further demonstrate that these two effects are uncoupled.

## DISCUSSION

The results shown in this study validate our yeast model of GAr-dependent translation inhibition, a mechanism that underlies EBV’s ability to evade the immune system. First, there is a strong correlation between the ability of the GAr domain of EBNA1 to inhibit translation in a *cis*- and length-dependent manner in yeast and human cells. Second, the Papio-30GAr domain is inactive in both yeast and human cells. Third, the yeast assay allowed the isolation of DXR, a drug found to increase the expression of GAr-containing proteins in a similar manner in yeast and human cells, and which is specifically able to increase the antigen presentation of peptides derived from the 235GAr-Ova fusion protein. Fourth, in the first corpus of SAR study performed with close chemical analogs of DXR, we observed a direct correlation between results obtained in the yeast and in the T-cell assays. Hence, the results presented here indicate that the cellular mechanism(s) involved in GAr-mediated inhibition of mRNA translation in *cis* are conserved from yeast to human. Our results also validate the use of the yeast-based assay to identify compounds potentially active in making EBV-infected cells targets for the immune system. In addition, our yeast model can also be used to perform genetic screens aimed at identifying cellular factors that contribute to the GAr-dependent translation control. The latter can also be achieved using reverse screening strategies to identify the conserved intracellular targets of the isolated active drugs (Gug et al., 2010; Guiffant et al., 2007; Tribouillard-Tanvier et al., 2008).

DXR is long-known to target topoisomerase-II–DNA complexes, thereby being genotoxic and used to treat some cancers. However, because our results show that the genotoxic effect of DXR and various analogs thereof is uncoupled from the effect on GAr-dependent inhibition of translation, they suggest that non-genotoxic approaches to target GAr-dependent translation control are feasible. In support of this, we observed that the DNA-damaging drug 5FU and the topoisomerase-II inhibitors etoposide and teniposide do not affect the GAr-dependent mRNA translation suppression.

The development of tumor-specific therapies is of highest priority for novel cancer treatments. Altogether, EBV-related cancers might represent 2–3% of total cancers, with important regional variations (Hsu and Glaser, 2000). In particular, nasopharyngeal carcinoma is one of the most frequent cancers affecting men in the South-East of China. Because all EBV-infected cells express the EBV-encoded EBNA1 protein, this offers a unique opportunity for the development of original immunotherapies involving specific targeting of virus-infected cells without damaging normal tissues. This study constitutes a proof of concept that our yeast-based model can be used to isolate drugs that can interfere with EBNA1-based EBV stealthiness, and encourages further screening of large and diverse compound libraries to identify leads for the development of new therapeutics specifically targeting EBV-infected tumor cells. Of note, EBV’s main targets are B cells, but this virus can also infect other cell types, including T cells and epithelial cells (Borza and Hutt-Fletcher, 2002). Importantly, whereas most tumor cells from EBV-related cancers are infected by EBV and express EBNA1, EBV latency in healthy carriers is primarily restricted to a specific pool of memory B cells and viral replication is spontaneously activated in only a small percentage of these cells (Fries et al., 1997). Therefore, treating EBV-related cancers by specifically interfering with EBV stealthiness might not present toxicity for non-tumoral cells.

The data presented here focus on EBV and EBNA1 but, based on published literature, it is possible that a similar strategy could also be applied to the LANA1 protein of the Kaposi’s-sarcoma-associated herpesvirus (KSHV; also named HHV-8 for human herpesvirus-8) (Kwun et al., 2007). Similar to EBV, KSHV has the ability to establish a latent infection in lymphoid cells and to induce cellular proliferation. Similarly to the GAr domain of EBNA1, the QED-rich central region of LANA1 has been involved in inhibition of the synthesis of its own protein and in KSHV immune evasion. Hence, our EBV-based approach could pave the way for new therapeutic approaches targeting tumor cells associated with other viruses such as KSHV.

Mainly based on antibiotics, drugs targeting ribosomal activity have been viewed as general inhibitors of protein synthesis, and regulation of the translation of specific mRNAs by therapeutic intervention has been considered less likely (Hermann, 2005). This notion is challenged by the results presented here, which, instead, indicate that the translation of at least certain viral mRNAs can specifically be targeted for therapeutic intervention. Furthermore, the translation of eukaryotic mRNAs displays a surprising specificity and inherited diseases with tissue-specific symptoms have been found to originate from defects in translation factors that were thought to have ubiquitous functions (Scheper et al., 2007). By understanding the mechanisms of mRNA translation in detail, it is likely that target opportunities will arise that can, as for the GAr domain, be exploited for assay development and compound screening aimed at controlling the translation of specific mRNAs.

## MATERIALS AND METHODS

### Yeast strains and culture media

All the yeast strains used in this study are derived from the W303 *WT* strain (Blondel et al., 2005): *MATa*, *leu2-3,112 trp1-1 can1-100 ura3-1 ade2-1 his3-11,15*. The *ade2Δ* strain genotype is: *MATa*, *leu2-3,112 trp1-1 can1-100 ura3-1 ade2-1::his5^S. pombe^*. Yeast cells were grown and used as previously described (Bach et al., 2003; Bach et al., 2006). The media used for yeast growth were: YPD [1% (w/v) yeast extract, 2% (w/v) peptone, 2% (w/v) glucose], ½ YPD [0.5% (w/v) yeast extract, 2% (w/v) peptone, 2% (w/v) glucose]. Solid media contained 2% (w/v) agar.

### Plasmid constructions

All plasmids were generated using standard procedures. Restriction enzymes, T4 DNA ligase and calf intestinal alkaline phosphatase were obtained from New England Biolabs (Ipswich, MA). Purified synthetic oligonucleotides were obtained from Eurogentec. Routine plasmid maintenance was carried out in DH5α and TOP10 bacteria strains. p416-*pADH-HA*-*ADE2* plasmid was constructed as follows: *HA*-*ADE2* was amplified using the sense primer 5′-GCGCGAATTCATGTACCCATACGATGTTCCAGATTACGCTAGGGATTCTAGAACAG-3′ and the antisense primer 5′-GCGCGGTACCTTACTTGTTTTCTAGATAAGG-3′ that introduced *Eco*RI and *Kpn*I restriction sites and was then cloned into p416-*pADH* vector. To construct p416-*pADH*-*HA-21GAr-ADE2* plasmid, *HA-21GAr* was amplified using the sense primer 5′-GCGCGGATCCATGGGGTACCCATACGATGTT-3′ and the antisense primer 5′-GCGCGAATTCTGGTGAATTCAGGGCCCCTCC-3′ that introduced *Bam*HI and *Eco*RI restriction sites and was then cloned into p416-*pADH-HA-ADE2* vector upstream of the *ADE2* gene. p416-*pADH*-*HA-43GAr-ADE2* plasmid was constructed in the same way and using the same primers as for p416-*pADH*-*HA-21GAr-ADE2* plasmid. p416-*pADH*-*HA-235GAr-ADE2* plasmid was constructed as follows: *HA-235GAr* was amplified using the sense primer 5′-GCGCGGATCCATGTACCCCTACGACGTCCCCG-3′ and the antisense primer 5′-GCGCGAATTCCTCGAGGATATCACCTTCTTGG-3′ that introduced *Bam*HI and *Eco*RI restriction sites and was then cloned into p416-*pADH-ADE2* vector upstream of the *ADE2* gene. To construct p416-*pADH*-*HA-papio30GAr-ADE2*, a *HA-papio30GAr* cassette was amplified using the sense primer 5′-GCGCGGATCCATGGCTTACCCCTACGACG-3′ and the antisense primer 5′-GCGCGGTGAATTCTCCTCCTGCTCC-3′ that introduced *Bam*HI and *Eco*RI restriction sites and was then cloned into the p416-*pADH-ADE2* plasmid upstream of the *ADE2* gene. To construct p416-*pTEF*-*HA-ADE2* plasmid, p416-*pADH*-*HA-ADE2* vector was digested with *Eco*RI and *Kpn*I and the released *HA-ADE2* cassette was inserted into p416-*pTEF* vector. To construct p416-*pTEF*-*HA-21GAr-ADE2*, p416-*pTEF*-*HA-43GAr-ADE2* and p416-*pTEF*-*HA-235GAr-ADE2* vectors, the *HA-21GAr-ADE2*, *HA-43GAr-ADE2* and *HA-235GAr-ADE2* cassettes were obtained by digesting the respective p416-*pADH* constructs with *Bam*HI and *Kpn*I, and then inserted into p416-*pTEF* vector. pCDNA3-*OVA*, pCDNA3-*235GAr-OVA*, pCDNA3-*EBNA1* and pCDNA3-*EBNA1ΔGAr* constructs were obtained as described previously (Apcher et al., 2010; Yin et al., 2003).

### Yeast strain constructions

In order to obtain a cell-to-cell homogeneous expression, we first stably integrated a single copy of *HA-43GAr-ADE2* or *HA-ADE2* under the control of the *ADH* promoter into the genome of an *ade2Δ* strain. *pADH-HA-ADE2*, *pADH-HA-21GAr-ADE2*, *pADH-HA-43GAr-ADE2* and *pTEF-HA-235GAr-ADE2* fragments were excised from p416-*pADH-HA-ADE2*, p416-*pADH-HA-21GAr-ADE2*, p416-*pADH-HA-43GAr-ADE2* and p416-*pTEF-HA-235GAr-ADE2* plasmids, respectively, using *Sac*I and *Kpn*I restriction enzymes, and sub-cloned into the disintegrator plasmid pIS375 (Sadowski et al., 2007). The disintegrator plasmids allow us to obtain a single-copy integration of the constructs of interest at the junction of the marker deletion, and the complete removal of additional plasmid sequences (Sadowski et al., 2007). Because the integrated constructs do not contain any flanking sequence duplication, the integrations are highly stable. Yeast transformations were performed using the lithium acetate procedure. The strains obtained are named *pADH-HA-ADE2*, *pADH-HA-21GAr-ADE2*, *pADH-HA-43GAr-ADE2* and *pTEF-HA-235GAr-ADE2*.

### Drugs and chemical libraries

Approximately 2000 compounds were screened from two different chemical libraries, including the Prestwick Chemical Library®, a collection of 1200 compounds at least in Phase II of clinical trials, and the BIOMOL’s FDA Approved Drug Library (Enzo Life Sciences), which consists of a collection of 640 FDA-approved drugs selected to maximize the chemical and pharmacological diversity. The compounds were supplied in 96-well plates as 10 mM (Prestwick®) and 2 mg/ml (BIOLMOL®) DMSO solutions. 5FU, DXR, daunorubicin quinacrine and methylene blue were purchased from Sigma-Aldrich and resuspended in DMSO. Epirubicin, idarubicin, pirarubicin, etoposide and teniposide were purchased from Santa Cruz, and valrubicin from Chemos.

### Yeast-based screening assay

This assay was adapted from an existing test (Bach et al., 2003; Bach et al., 2006; Tribouillard et al., 2006). An aliquot of an exponentially growing culture (200 μl of a 0.55 OD_600_ culture) of the *pADH*-*HA-43GAr-ADE2* strain was spread homogeneously using sterile glass beads (3 and 5 mm diameter) on square (12 cm × 12 cm) Petri dishes containing YPD solid medium. Small sterile filters (Thermo Fisher) were then placed on the agar surface. 2 μl of individual compounds from various chemical libraries were applied to each filter in addition to the top left filter where DMSO, the vehicle, was added as a negative control. Plates were then incubated at 29°C for 4–5 days and scanned using a Snap Scan1212 (Agfa).

### Yeast cell culture

For liquid cultures, *pADH-HA-43GAr-ADE2* exponentially growing yeast cells cultured in YPD at 29°C were plated onto six-well plates (10 μl, OD_600_=0.5 per well) poured with YPD solid medium containing 5FU or DXR at indicated concentrations for 5 days. For color determination, cells were harvested in 1 ml of minimum medium MML (Difco) and placed in 2-ml plastic cuvettes, which were photographed. To analyze protein content, cells grown on six-well plates were harvested in YPD and lysed. Proteins were analyzed by western blotting as described below.

### Cell lines and culture conditions

HEK 293T Kb cells originate from human embryonic kidney cells and stably express the MHC class I Kb molecule. Raji cells are type III latency Burkitt’s lymphoma and A549 are lung-carcinoma-derived cells expressing wild-type endogenous p53. The SIINFEKL/Kb-specific (B3Z) CD8^+^ T-cell hybridoma (Shastri and Gonzalez, 1993) expresses *lacZ* in response to the activation of T-cell receptors specific for the SIINFEKL peptide (Ova-immunodominant peptide) in the context of H-2Kb (Kb) MHC class I molecules. The human HEK 293 T Kb stable cell line was obtained from Dr L. Eisenlohr (Thomas Jefferson University, Philadelphia, PA). The cell lines (HEK-293T Kb, B3Z and Raji) were cultured under standard conditions in DMEM or RPMI 1640 media supplemented with 10% heat-inactivated fetal calf serum, 2 mM L-glutamine and 100 IU/ml penicillin and streptomycin (Gibco-BRL).

### Protein extracts and western blot analysis

For yeast protein extracts, a total of 10 ml of a 0.6 OD_600_ culture of exponentially growing cells were harvested by centrifugation and cell pellets were resuspended in lysis buffer [25 mM Tris-HCl, pH 7.4, 100 mM NaCl, 0.2% Triton X-100, antiproteases cocktail (Roche), 1 mM phenyl-methylsulfonyl fluoride]. After addition of 425-600 μm glass beads (Sigma-Aldrich), cells were lysed by vortexing for 30 seconds followed by 30 seconds ice-cooling for six times and then centrifuged for 3 minutes at 200 ***g*** at 4°C. Supernatants were recovered and assayed for protein content. Following heat denaturation for 3 minutes at 95°C, protein extracts were analyzed by 10% SDS-PAGE (precast NuPAGE, Invitrogen) and transferred onto 0.45-μm nitrocellulose membranes (Whatman). Membranes were blocked with PBS 1×/0.1% Igepal containing 5% fat-free milk and 0.5% BSA, and incubated overnight at 4°C with the indicated primary antibodies (mouse anti-HA, mouse anti-Actin IgM, Calbiochem). The membranes were then washed with fresh PBS 1×/0.1% Igepal and incubated for 45 minutes with secondary antibodies (goat anti-mouse, Bio-Rad; goat anti-mouse IgM, Calbiochem) conjugated to horseradish peroxidase at a 1:3000 dilution, and analyzed by enhanced chemiluminescence (ECL, Amersham) using a Vilber-Lourmat Photodocumentation Chemistart 5000 imager. The mouse anti-HA serum was a gift from B. Vojtesek (RECAMO, Brno, Czech Republic) and was used at a 1:5000 dilution, as was the anti-actin antibody. For western blot analysis of mammalian proteins, cells were harvested 48 hours post-transfection and lysed in lysis buffer (20 mM Tris, pH 7.5, 150 mM NaCl, 1% NP40) containing protease inhibitors (Roche, Germany). Protein concentrations were measured using a Bradford assay. Total cell extracts were fractionated by SDS-PAGE, transferred to BioTrace NT Nitrocellulose Blotting membranes (PALL) and probed with anti-Ova rabbit polyclonal antibody (Sigma) or anti-EBNA1 mouse monoclonal antibody (OT1X), both at a 1:1000 dilution, or anti-p53 antibody (DO1, 1:1000), or anti-p21 antibody (12D1, Cell Signaling, 1:500). After incubation with the appropriate horseradish-peroxidase-conjugated secondary antibodies, proteins were visualized by ECL (Amersham).

### Pulse-chase assay

Exponentially growing *pADH-HA-ADE2* or *pADH-HA-43GAr-ADE2* yeast cells were pulse-labeled with 90 μCi/OD_600_ (EasyTag Express protein labeling mix [^35^S], PerkinElmer Life Sciences) for 15 minutes after being cultured in methionine-free medium for 15 minutes, and then chased in fresh medium containing 50 mM cold methionine and cysteine for the indicated time points before harvesting. After centrifugation, cell pellets were resuspended in lysis buffer [25 mM Tris-HCl, pH 7.4, 100 mM NaCl, 0.2% Triton X-100, antiprotease cocktail (Roche), 1 mM phenyl-methylsulfonyl fluoride] and treated as described above. Lysates were pre-cleared with protein G-Sepharose beads for 45 minutes at 4°C and further immunoprecipitated with 1 μg of anti-HA monoclonal antibodies pre-bound to protein G-Sepharose beads overnight at 4°C. The beads were then washed with PBS 1×/0.2% Igepal four times and boiled in SDS loading buffer. Immunoprecipitates were analyzed by SDS-PAGE using 10% precast NUPAGE gels (Invitrogen). The gel was dried and analyzed using a Typhoon 9400 Phosphorimager (GE).

### Quantitative real-time PCR

Total yeast cellular RNA was extracted using RNAeasy and RNase-free DNase kits (QIAGEN). cDNA synthesis was carried out from 1 μg of DNA-free RNA using Superscript II (Invitrogen) using random hexamers (Qiagen). Duplicate cDNA samples were subjected to quantitative PCR using QuantiTect SYBR Green PCR kit (Qiagen) using the Abiprism 7000 Sequence Detection System (Applied). The relative abundance of amplified mRNA was determined, using actin as a control for normalization. The primer pairs used for PCR were as follows: *ADE2*-forward: 5′-ATTGTGCAAATGCCTAGAGGTG-3′, *ADE2*-reverse: 5′-AATCATAAGCGCCAAGCAGTC-3′, Actin-forward: 5′-ATGGTNGGNATGGGNCARAAR-3′, Actin-reverse: 5′-YTCCATRTCRTCCCAGTTGGT-3′. In these sequences, ‘R’ is for purine (A or G), ‘Y’ is for pyrimidine (C or T) and ‘N’ is for any nucleotide.

### T-cell assay

This assay was performed as previously described (Apcher et al., 2010). Briefly, HEK 293T Kb cells were seeded in six-well plates at a density of 200,000 cells/well. The following day, cells were transfected with 1 μg of *OVA* or *235GAr-OVA* expression plasmids with 3 μl of Genejuice according to the manufacturer’s protocol (Merck Biosciences). The day after, transfected cells were treated with increasing concentrations of drugs. 24 hours later, transfected and treated HEK 293T Kb cells were plated at a density of 50,000 cells/well in 96-well plates and cultured in the presence of 50,000 cells/well of B3Z T-cell hybridoma for 16 hours. Cells were then harvested and washed twice with 1× cold PBS prior to lysis in 0.2% Triton X-100, 0.5 M K_2_HPO_4_, 0.5 M KH_2_PO_4_ for 5 minutes on ice. Cell lysates were centrifuged for 10 minutes at 200 ***g***, and 25 μl of supernatant from each well were transferred into 96-well Optiplate counting plates (Packard Bioscience, Randburg, SA) and tested for β-galactosidase activity using a Luminescence assay (BD Biosciences, Clontech) on a Fluorostar reader. The results were expressed as arbitrary Gal units and data were normalized to the presentation of the exogenous SIINFEKL (SL8) peptide (corresponding to ovalbumin amino acids 257–264), which was purchased from Eurogentec (Seraing, Belgium).

### Polysome profiling

HEK 293T Kb cells were transfected with Ova or 235GAr-Ova expression plasmids. Cells expressing Ova or GAr-Ova were treated with DMSO or 1 μM doxorubicin for 16 hours. Cells were lysed in Polysomal Lysis Buffer (300 mM KCl, 5 mM MgCl_2_, 10 mM Hepes pH 7.4 and 0.5% NP40 in the presence of 0.1 mg/ml of cycloheximide and RNAase out) and analyzed on a 15–45% sucrose gradient (15 mM Tris-HCl, pH 7.5, 0.3 M KCl, 15 mM MgCl_2_, 1 mM dithiothreitol in the presence of 0.1 mg/ml of cycloheximide). Ultracentrifugation was performed using a SW41 rotor at 35,000 rpm for 3h 20 min at 4°C. Gradients were analyzed using a WellChrom Filter-Photometer K-2001UV and Brandel top collecting pump system at OD_254_. RNA purification from the 12 sucrose fractions of 1 ml collected for each sucrose gradient was performed using ethanol precipitation and RNeasy Mini Kit (Qiagen). Reverse transcription was carried out using equal volume of DNA-free RNA from each fraction with the M-MLV reverse transcriptase and the oligo-dT primer (Invitrogen). Step-One real-time PCR system was used for quantitative RT-PCR and the reaction was performed with the PerfectaSYBR green Fast mix ROX (Quanta). The primers used (forward and reverse, respectively) are: 5′-GAGGAGGCTTGGAACCTAT-3′ and 5′-CAGTTTGAGAATCCACGGAG-3′. The relative mRNA level of each fraction was calculated as a percentage of the total mRNA levels from all the fractions.

## Supplementary Material

Supplementary Material

## References

[b1-0070435] Apcher S. Komarova A. Daskalogianni C. Yin Y. Malbert-Colas L. Fåhraeus R.(2009). mRNA translation regulation by the Gly-Ala repeat of Epstein-Barr virus nuclear antigen 1. J. Virol. 83, 1289–1298 19019958 10.1128/JVI.01369-08 PMC2620890

[b2-0070435] Apcher S. Daskalogianni C. Manoury B. Fåhraeus R. (2010). Epstein Barr virus-encoded EBNA1 interference with MHC class I antigen presentation reveals a close correlation between mRNA translation initiation and antigen presentation. PLoS Pathog. 6, e1001151 20976201 10.1371/journal.ppat.1001151 PMC2954899

[b3-0070435] Apcher S. Daskalogianni C. Lejeune F. Manoury B. Imhoos G. Heslop L. Fåhraeus R. (2011). Major source of antigenic peptides for the MHC class I pathway is produced during the pioneer round of mRNA translation. Proc. Natl. Acad. Sci. USA 108, 11572–11577 21709220 10.1073/pnas.1104104108 PMC3136330

[b4-0070435] Apcher S. Manoury B. Fåhraeus R. (2012). The role of mRNA translation in direct MHC class I antigen presentation. Curr. Opin. Immunol. 24, 71–76 22341517 10.1016/j.coi.2012.01.007

[b5-0070435] Bach S. Talarek N. Andrieu T. Vierfond J. M. Mettey Y. Galons H. Dormont D. Meijer L. Cullin C. Blondel M. (2003). Isolation of drugs active against mammalian prions using a yeast-based screening assay. Nat. Biotechnol. 21, 1075–1081 12910243 10.1038/nbt855

[b6-0070435] Bach S. Tribouillard D. Talarek N. Desban N. Gug F. Galons H. Blondel M. (2006). A yeast-based assay to isolate drugs active against mammalian prions. Methods 39, 72–77 16750390 10.1016/j.ymeth.2006.04.005

[b7-0070435] Bilsland E. Sparkes A. Williams K. Moss H. J. de Clare M. Pir P. Rowland J. Aubrey W. Pateman R. Young M. (2013). Yeast-based automated high-throughput screens to identify anti-parasitic lead compounds. Open Biol 3, 120158 23446112 10.1098/rsob.120158 PMC3603448

[b8-0070435] Blake N. (2010). Immune evasion by gammaherpesvirus genome maintenance proteins. J. Gen. Virol. 91, 829–846 20089802 10.1099/vir.0.018242-0

[b9-0070435] Blake N. Lee S. Redchenko I. Thomas W. Steven N. Leese A. Steigerwald-Mullen P. Kurilla M. G. Frappier L. Rickinson A. (1997). Human CD8+ T cell responses to EBV EBNA1: HLA class I presentation of the (Gly-Ala)-containing protein requires exogenous processing. Immunity 7, 791–802 9430224 10.1016/s1074-7613(00)80397-0

[b10-0070435] Blondel M. (2012). Flirting with CFTR modifier genes at happy hour. Genome Med 4, 98 23270638 10.1186/gm399 PMC3580438

[b11-0070435] Blondel M. Bach S. Bamps S. Dobbelaere J. Wiget P. Longaretti C. Barral Y. Meijer L. Peter M. (2005). Degradation of Hof1 by SCF(Grr1) is important for actomyosin contraction during cytokinesis in yeast. EMBO J. 24, 1440–1452 15775961 10.1038/sj.emboj.7600627 PMC1142548

[b12-0070435] Borza C. M. Hutt-Fletcher L. M. (2002). Alternate replication in B cells and epithelial cells switches tropism of Epstein-Barr virus. Nat. Med. 8, 594–599 12042810 10.1038/nm0602-594

[b13-0070435] Couplan E. Aiyar R. S. Kucharczyk R. Kabala A. Ezkurdia N. Gagneur J. St Onge R. P. Salin B. Soubigou F. Le Cann M. (2011). A yeast-based assay identifies drugs active against human mitochondrial disorders. Proc. Natl. Acad. Sci. USA 108, 11989–11994 21715656 10.1073/pnas.1101478108 PMC3141935

[b14-0070435] Cristillo A. D. Mortimer J. R. Barrette I. H. Lillicrap T. P. Forsdyke D. R. (2001). Double-stranded RNA as a not-self alarm signal: to evade, most viruses purine-load their RNAs, but some (HTLV-1, Epstein-Barr) pyrimidine-load. J. Theor. Biol. 208, 475–491 11222051 10.1006/jtbi.2000.2233

[b15-0070435] Epstein M. A. Barr Y. M. Achong B. G. (1964). A Second Virus-Carrying Tissue Culture Strain (Eb2) of Lymphoblasts from Burkitt’s Lymphoma. Pathol. Biol. (Paris) 12, 1233–1234 14255814

[b16-0070435] Fåhraeus R. (2005). Do peptides control their own birth and death? Nat. Rev. Mol. Cell Biol. 6, 263–267 15738990 10.1038/nrm1590

[b17-0070435] Fries K. L. Sculley T. B. Webster-Cyriaque J. Rajadurai P. Sadler R. H. Raab-Traub N. (1997). Identification of a novel protein encoded by the BamHI A region of the Epstein-Barr virus. J. Virol. 71, 2765–2771 9060630 10.1128/jvi.71.4.2765-2771.1997 PMC191399

[b18-0070435] Giorgini F. Guidetti P. Nguyen Q. Bennett S. C. Muchowski P. J. (2005). A genomic screen in yeast implicates kynurenine 3-monooxygenase as a therapeutic target for Huntington disease. Nat. Genet. 37, 526–531 15806102 10.1038/ng1542 PMC1449881

[b19-0070435] Gruhne B. Sompallae R. Marescotti D. Kamranvar S. A. Gastaldello S. Masucci M. G. (2009). The Epstein-Barr virus nuclear antigen-1 promotes genomic instability via induction of reactive oxygen species. Proc. Natl. Acad. Sci. USA 106, 2313–2318 19139406 10.1073/pnas.0810619106 PMC2650153

[b20-0070435] Gug F. Oumata N. Tribouillard-Tanvier D. Voisset C. Desban N. Bach S. Blondel M. Galons H. (2010). Synthesis of conjugates of 6-aminophenanthridine and guanabenz, two structurally unrelated prion inhibitors, for the determination of their cellular targets by affinity chromatography. Bioconjug. Chem. 21, 279–288 20092293 10.1021/bc900314n

[b21-0070435] Guiffant D. Tribouillard D. Gug F. Galons H. Meijer L. Blondel M. Bach S. (2007). Identification of intracellular targets of small molecular weight chemical compounds using affinity chromatography. Biotechnol. J. 2, 68–75 17225251 10.1002/biot.200600223

[b22-0070435] Heessen S. Dantuma N. P. Tessarz P. Jellne M. Masucci M. G. (2003). Inhibition of ubiquitin/proteasome-dependent proteolysis in Saccharomyces cerevisiae by a Gly-Ala repeat. FEBS Lett. 555, 397–404 14644450 10.1016/s0014-5793(03)01296-1

[b23-0070435] Hermann T. (2005). Drugs targeting the ribosome. Curr. Opin. Struct. Biol. 15, 355–366 15919197 10.1016/j.sbi.2005.05.001

[b24-0070435] Hsu J. L. Glaser S. L. (2000). Epstein-barr virus-associated malignancies: epidemiologic patterns and etiologic implications. Crit. Rev. Oncol. Hematol. 34, 27–53 10781747 10.1016/s1040-8428(00)00046-9

[b25-0070435] Kapoor P. Frappier L. (2003). EBNA1 partitions Epstein-Barr virus plasmids in yeast cells by attaching to human EBNA1-binding protein 2 on mitotic chromosomes. J. Virol. 77, 6946–6956 12768013 10.1128/JVI.77.12.6946-6956.2003 PMC156160

[b26-0070435] Kapoor P. Shire K. Frappier L. (2001). Reconstitution of Epstein-Barr virus-based plasmid partitioning in budding yeast. EMBO J. 20, 222–230 11226172 10.1093/emboj/20.1.222 PMC140207

[b27-0070435] Karttunen J. Sanderson S. Shastri N. (1992). Detection of rare antigen-presenting cells by the lacZ T-cell activation assay suggests an expression cloning strategy for T-cell antigens. Proc. Natl. Acad. Sci. USA 89, 6020–6024 1378619 10.1073/pnas.89.13.6020 PMC402130

[b28-0070435] Kennedy G. Komano J. Sugden B. (2003). Epstein-Barr virus provides a survival factor to Burkitt’s lymphomas. Proc. Natl. Acad. Sci. USA 100, 14269–14274 14603034 10.1073/pnas.2336099100 PMC283581

[b29-0070435] Kwun H. J. da Silva S. R. Shah I. M. Blake N. Moore P. S. Chang Y. (2007). Kaposi’s sarcoma-associated herpesvirus latency-associated nuclear antigen 1 mimics Epstein-Barr virus EBNA1 immune evasion through central repeat domain effects on protein processing. J. Virol. 81, 8225–8235 17522213 10.1128/JVI.00411-07 PMC1951309

[b30-0070435] Levitskaya J. Coram M. Levitsky V. Imreh S. Steigerwald-Mullen P. M. Klein G. Kurilla M. G. Masucci M. G. (1995). Inhibition of antigen processing by the internal repeat region of the Epstein-Barr virus nuclear antigen-1. Nature 375, 685–688 7540727 10.1038/375685a0

[b31-0070435] Louie R. J. Guo J. Rodgers J. W. White R. Shah N. Pagant S. Kim P. Livstone M. Dolinski K. McKinney B. A. (2012). A yeast phenomic model for the gene interaction network modulating CFTR-ΔF508 protein biogenesis. Genome Med 4, 103 23270647 10.1186/gm404 PMC3906889

[b32-0070435] Mager W. H. Winderickx J. (2005). Yeast as a model for medical and medicinal research. Trends Pharmacol. Sci. 26, 265–273 15860374 10.1016/j.tips.2005.03.004

[b33-0070435] Nitiss J. L. (2009). Targeting DNA topoisomerase II in cancer chemotherapy. Nat. Rev. Cancer 9, 338–350 19377506 10.1038/nrc2607 PMC2748742

[b34-0070435] Perocchi F. Mancera E. Steinmetz L. M. (2008). Systematic screens for human disease genes, from yeast to human and back. Mol. Biosyst. 4, 18–29 18075670 10.1039/b709494a

[b35-0070435] Rickinson A. B. Moss D. J. (1997). Human cytotoxic T lymphocyte responses to Epstein-Barr virus infection. Annu. Rev. Immunol. 15, 405–431 9143694 10.1146/annurev.immunol.15.1.405

[b36-0070435] Rowe M. Rowe D. T. Gregory C. D. Young L. S. Farrell P. J. Rupani H. Rickinson A. B. (1987). Differences in B cell growth phenotype reflect novel patterns of Epstein-Barr virus latent gene expression in Burkitt’s lymphoma cells. EMBO J. 6, 2743–2751 2824192 10.1002/j.1460-2075.1987.tb02568.x PMC553698

[b37-0070435] Sadowski I. Su T. C. Parent J. (2007). Disintegrator vectors for single-copy yeast chromosomal integration. Yeast 24, 447–455 17315265 10.1002/yea.1469

[b38-0070435] Scheper G. C. van der Knaap M. S. Proud C. G. (2007). Translation matters: protein synthesis defects in inherited disease. Nat. Rev. Genet. 8, 711–723 17680008 10.1038/nrg2142

[b39-0070435] Shastri N. Gonzalez F. (1993). Endogenous generation and presentation of the ovalbumin peptide/Kb complex to T cells. J. Immunol. 150, 2724–2736 8454852

[b40-0070435] Starck S. R. Ow Y. Jiang V. Tokuyama M. Rivera M. Qi X. Roberts R. W. Shastri N. (2008). A distinct translation initiation mechanism generates cryptic peptides for immune surveillance. PLoS ONE 3, e3460 18941630 10.1371/journal.pone.0003460 PMC2565129

[b41-0070435] Tellam J. Smith C. Rist M. Webb N. Cooper L. Vuocolo T. Connolly G. Tscharke D. C. Devoy M. P. Khanna R. (2008). Regulation of protein translation through mRNA structure influences MHC class I loading and T cell recognition. Proc. Natl. Acad. Sci. USA 105, 9319–9324 18591662 10.1073/pnas.0801968105 PMC2453702

[b42-0070435] Tellam J. T. Lekieffre L. Zhong J. Lynn D. J. Khanna R. (2012). Messenger RNA sequence rather than protein sequence determines the level of self-synthesis and antigen presentation of the EBV-encoded antigen, EBNA1. PLoS Pathog. 8, e1003112 23300450 10.1371/journal.ppat.1003112 PMC3531512

[b43-0070435] Thorley-Lawson D. A. Allday M. J. (2008). The curious case of the tumour virus: 50 years of Burkitt’s lymphoma. Nat. Rev. Microbiol. 6, 913–924 19008891 10.1038/nrmicro2015

[b44-0070435] Tribouillard D. Bach S. Gug F. Desban N. Beringue V. Andrieu T. Dormont D. Galons H. Laude H. Vilette D. (2006). Using budding yeast to screen for anti-prion drugs. Biotechnol. J. 1, 58–67 16892225 10.1002/biot.200500001

[b45-0070435] Tribouillard-Tanvier D. Dos Reis S. Gug F. Voisset C. Béringue V. Sabate R. Kikovska E. Talarek N. Bach S. Huang C. (2008). Protein folding activity of ribosomal RNA is a selective target of two unrelated antiprion drugs. PLoS ONE 3, e2174 18478094 10.1371/journal.pone.0002174 PMC2374897

[b46-0070435] Wilson J. B. Bell J. L. Levine A. J. (1996). Expression of Epstein-Barr virus nuclear antigen-1 induces B cell neoplasia in transgenic mice. EMBO J. 15, 3117–3126 8670812 PMC450254

[b47-0070435] Yewdell J. W. (2011). DRiPs solidify: progress in understanding endogenous MHC class I antigen processing. Trends Immunol. 32, 548–558 21962745 10.1016/j.it.2011.08.001 PMC3200450

[b48-0070435] Yin Y. Manoury B. Fåhraeus R. (2003). Self-inhibition of synthesis and antigen presentation by Epstein-Barr virus-encoded EBNA1. Science 301, 1371–1374 12958359 10.1126/science.1088902

[b49-0070435] Young L. S. Rickinson A. B. (2004). Epstein-Barr virus: 40 years on. Nat. Rev. Cancer 4, 757–768 15510157 10.1038/nrc1452

