# Supplementary material for: A yeast-based assay identifies drugs that interfere with immune evasion of the Epstein-Barr virus
Source: Dis Model Mech. 2014 Feb 20;7(4):435–44. doi: 10.1242/dmm.014308 (PMC3974454; doi:10.1242/dmm.014308)
Supplement: Supplementary Material [file supp_7_4_435__index.html]

A yeast-based assay identifies drugs that interfere with immune evasion of the Epstein-Barr virus — Supplementary Material 

# A yeast-based assay identifies drugs that interfere with immune evasion of the Epstein-Barr virus

## DMM014308 Supplementary Material

**Files in this Data Supplement:**

- **Supplementary Material**
