# Supplementary material for: A yeast-based assay identifies drugs that interfere with immune evasion of the Epstein-Barr virus
Source: Dis Model Mech. 2014 Feb 20;7(4):435–44. doi: 10.1242/dmm.014308 (PMC3974454; doi:10.1242/dmm.014308)
Supplement: Supplementary Material [file supp_7.4.435_DMM014308.pdf]

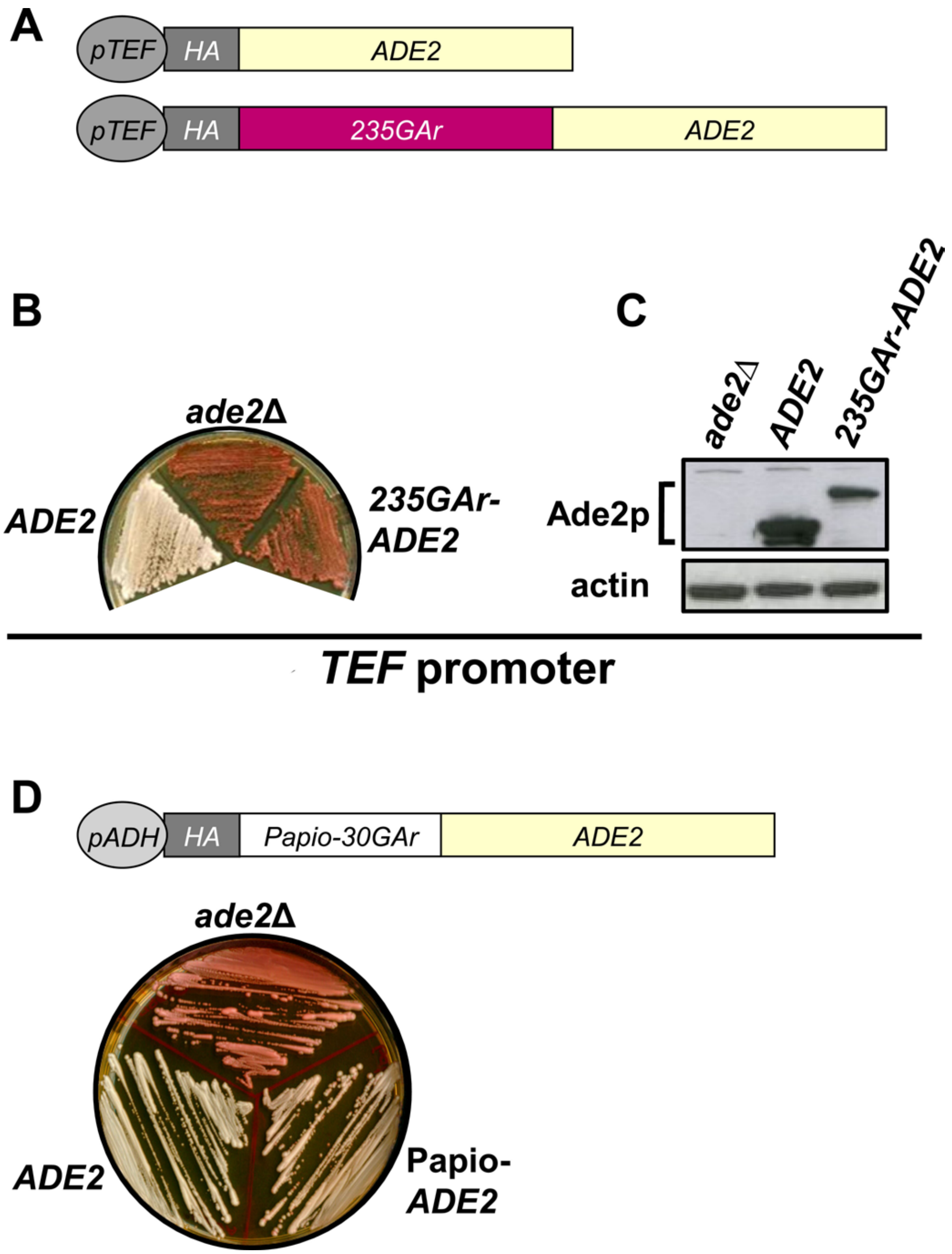

Supplementary Figure 1: The effect of the GAr domain on translation in yeast is promoter-independent and the *papio-30-GAr-ADE2* fusion

The full-length *235GAr* fused to the *ADE2* gene also inhibits the translation of its own mRNA when expressed from the strongest constitutive *TEF* promoter. **A**) The two constructs used are depicted: both are HA-tagged and expressed from the *TEF* promoter. **B**) Clones of *ade2Δ* strain, expressing, or not, the two constructs depicted in (**A**) were streaked on a glucose-rich medium and incubated for 5 days at 29°C. **C**) SDS-PAGE and Western blot analysis of extracts from cells used in (**B**). **D**) Clones of *ade2Δ* strain expressing, or not, *ADE2* or the *papio-30-ADE2* construct depicted above.

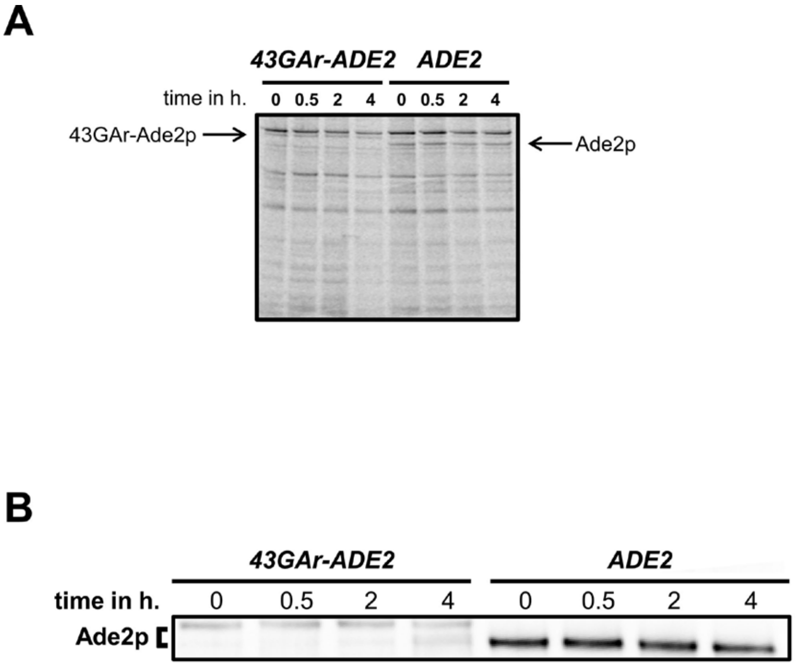

Supplementary Figure 2: Pulse-chase analysis showing that the 43GAr domain inhibits the synthesis of Ade2p but has no effect on its stability

Yeast cells expressing *HA-43GAr-ADE2* or *HA-ADE2* were incubated with a mixture of radio-labeled methionine/cysteine for 15 minutes (pulse), at which time a large excess of a mixture of non-labeled methionine/cysteine was added (chase). **A**) At the indicated time points, protein extracts were prepared, immunoprecipitated using an anti-HA antibody and then analyzed by SDS-PAGE followed by autoradiography using a Storm phosphorimager. **B**) The same immunoprecipitated proteins were analyzed by SDS-PAGE and Western blot using anti-HA antibodies.

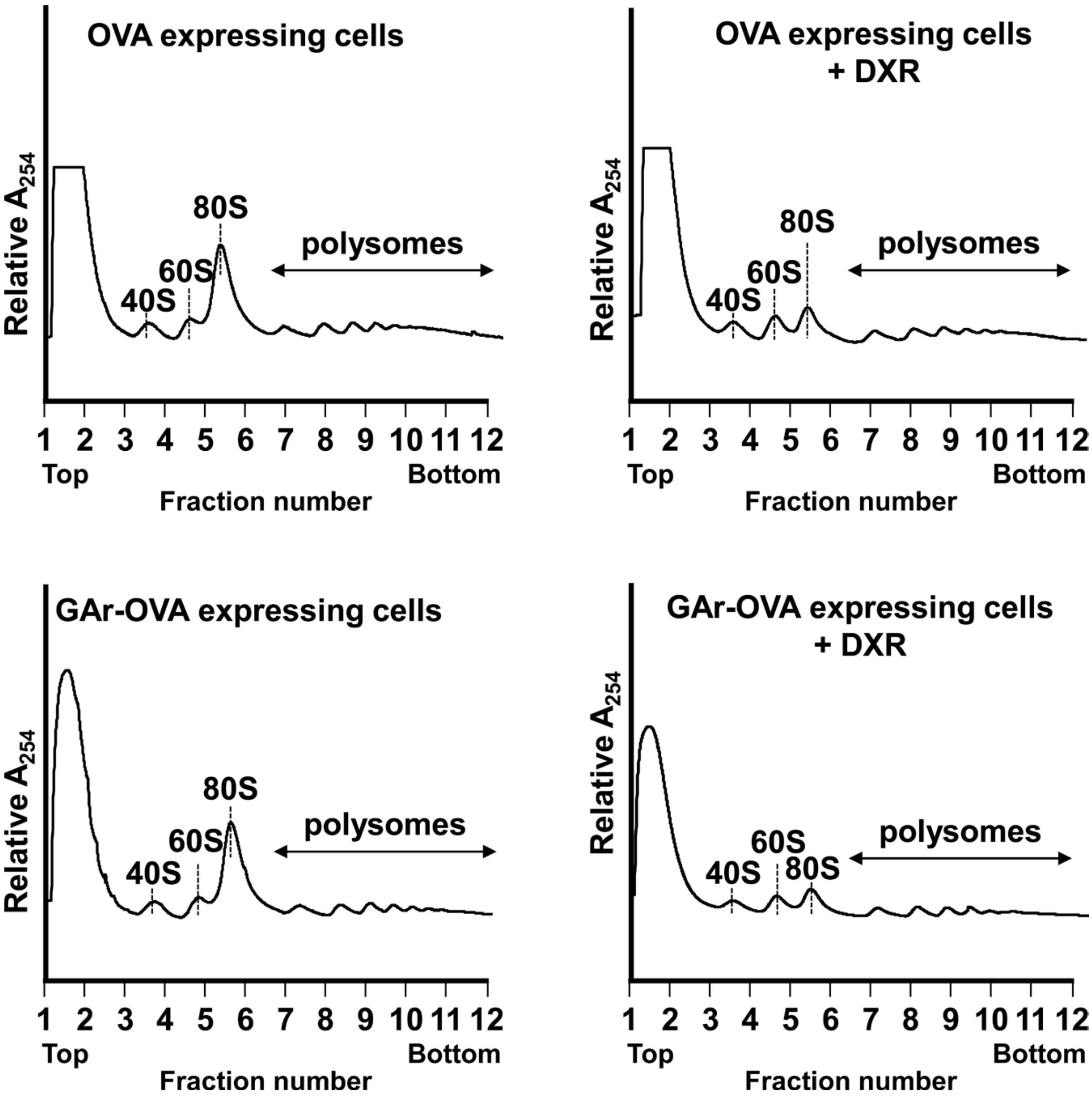

Supplementary Figure 3: polysome profiles of OVA and GAr-OVA expressing cells treated by DXR.
HEK 293T Kb cells expressing OVA or GAr-OVA treated with DMSO or 1 µM DXR for 16h.

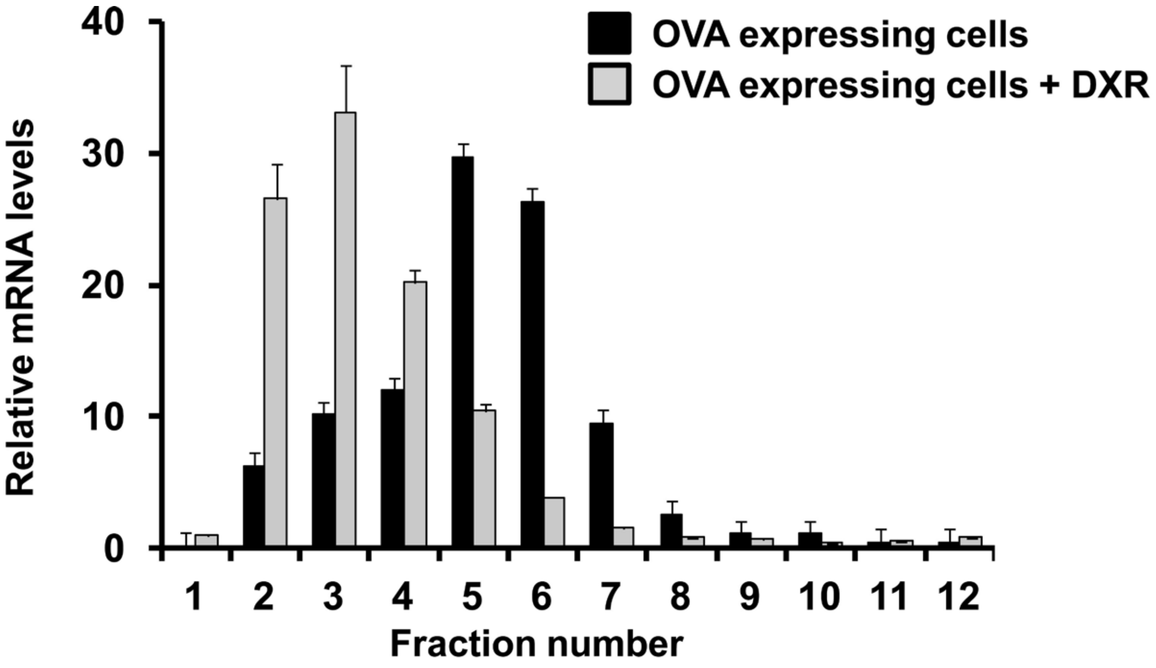

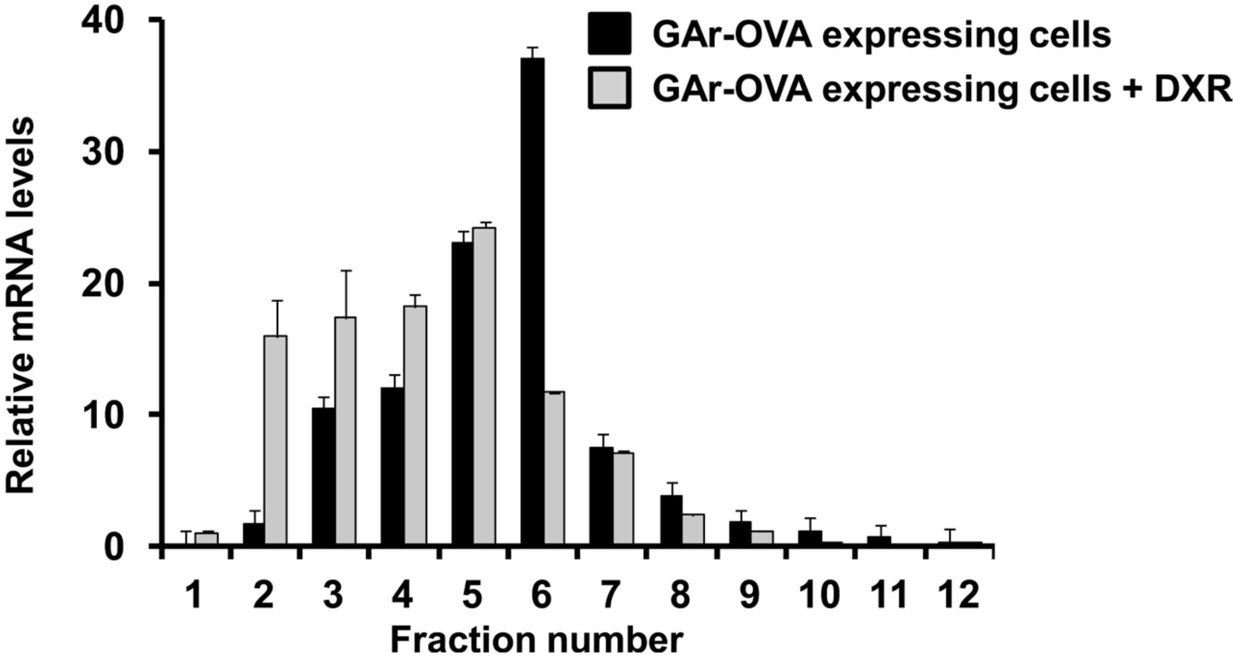

Supplementary Figure 4: DXR alters the interaction of ribosome with OVA and GAr-OVA mRNAs.
RNAs were extracted from the 12 sucrose fractions of polysome gradients shown in **Figure S3**. qRT-PCR was used to determine the relative levels of OVA and GAr-OVA mRNAs present in each polysome fraction. Relative OVA or GAr-OVA mRNA levels in each fraction were calculated as a percentage of the total mRNA levels from all the fractions.
